# Microfluidic Immunomagnetic Capture of Circulating Tumor Cells via On-Bead Antibody Conjugation

**DOI:** 10.7150/jca.134785

**Published:** 2026-06-25

**Authors:** Ming-Lin Tsai, Wen-Ching Hsieh, Sung-Chi Tsai, Yi-Chiung Hsu

**Affiliations:** 1 Department of General Surgery, Cathay General Hospital, Taipei 10630, Taiwan.; 2 Department of Biomedical Sciences and Engineering, National Central University, Taoyuan City 320317, Taiwan.; 3 Department of Medical Research, Cathay General Hospital, Taipei 10630, Taiwan.; 4 Center for Astronautical Physics and Engineering, National Central University, Taoyuan City 320317, Taiwan.

**Keywords:** microfluidic chip, circulating tumor cells (CTCs), immunomagnetic capture, magnetic bead-antibody conjugation

## Abstract

Microfluidic immunomagnetic platforms have been widely explored for circulating tumor cell (CTC) enrichment; however, operational complexity, unstable flow control, and limited real-time observation remain practical challenges for routine implementation. In this study, we developed an integrated microfluidic chip system for EpCAM-based immunomagnetic enrichment of tumor cells using antibody-functionalized magnetic beads. The platform incorporates a gas-liquid separation structure, pressure-regulated flow control, and a transparent chip design that enables real-time microscopic observation of bead-cell interactions during capture. Biotin loading on streptavidin-coated beads was optimized, with surface saturation observed at 6 nmol. Flow-rate optimization identified 0.4 μL/s as the selected flow rate, balancing capture efficiency and cell-bead interaction time. In whole-blood spike-in experiments using MCF-7 cells, the platform achieved recovery efficiencies ranging from approximately 57% to 91%, depending on the input cell number. Cells with Hoechst⁺/CD326⁺/CD45⁻ phenotypes were also observed in patient-derived blood samples, supporting the feasibility of detecting putative CTCs in clinical specimens. This study positions the system as an engineering-integrated, real-time-observable platform for CTC enrichment. Further validation using larger clinical cohorts, multi-marker capture strategies, quantitative purity analysis, and cell viability assays will be required before translational application.

## 1. Introduction

Circulating tumor cells (CTCs) are malignant cells shed from primary or metastatic tumors into the bloodstream and are widely recognized as key contributors to cancer metastasis and disease progression 1,2. As an emerging target for liquid biopsy, CTC analysis provides real-time and clinically relevant information for early cancer diagnosis, therapeutic monitoring, and prognosis evaluation 3,4. Compared with conventional invasive tissue biopsies, liquid biopsy offers significant advantages, including minimal invasiveness, repeatable sampling, and dynamic disease monitoring, thereby attracting considerable clinical and research interest 2,5. However, the extremely low abundance of CTCs in peripheral blood—typically only 1-10 CTCs per milliliter—together with their pronounced biological heterogeneity, poses substantial technical challenges for efficient capture and accurate analysis 6,7.

Currently developed CTC isolation technologies can be broadly categorized into approaches based on physical properties and those based on biological properties. Physical methods, such as size-based filtration and density-based separation, are relatively straightforward to implement but often suffer from limited specificity due to the overlapping physical characteristics of CTCs and leukocytes 1, leading to nonspecific capture and background interference 8,9. In contrast, biologically based immunoaffinity methods utilize the recognition of tumor-associated surface antigens, such as the epithelial cell adhesion molecule (EpCAM) 10,11, to achieve higher selectivity. Nevertheless, antigen expression heterogeneity arising from epithelial-mesenchymal transition (EMT) can significantly compromise capture efficiency and reproducibility 1,12.

In recent years, microfluidic technologies have emerged as promising platforms for CTC isolation and analysis owing to their low sample consumption, precise fluid manipulation, high surface-to-volume ratio, and strong potential for system integration 11. Through rational microchannel design, microfluidic systems can enhance the interaction probability between target cells and capture interfaces while operating under physiologically relevant flow conditions. Despite these advantages, many existing microfluidic CTC capture platforms still face limitations, including insufficient automation, complex operational procedures, and inconsistent capture performance across experiments, which collectively hinder their clinical translation 13,14.

Immunomagnetic capture strategies based on antibody-functionalized magnetic beads offer a practical approach to improve CTC isolation performance further 15. Magnetic beads conjugated with specific antibodies can selectively bind target tumor cells and enable selective manipulation and enrichment under external magnetic fields. When integrated with microfluidic platforms, magnetic bead-antibody conjugation not only enhances capture efficiency and specificity but also enables direct processing of whole blood samples, thereby minimizing extensive preprocessing steps that may adversely affect cell integrity and recovery 12,16.

Based on the advantages and limitations of current immunomagnetic and microfluidic CTC capture systems, this study presents a microfluidic chip platform that integrates pressure-regulated flow control with magnetic bead-antibody conjugation to enrich circulating tumor cells (CTCs) 17,18. The proposed system incorporates a gas-liquid separation structure to improve flow stability and minimize bubble-induced disturbance, while a transparent chip design enables real-time microscopic observation of bead-cell interactions during capture. In addition, antibody functionalization of magnetic beads was optimized to enhance binding performance under continuous-flow conditions. System performance was evaluated using spike-in experiments with cultured cancer cells in whole blood, followed by preliminary assessment using patient-derived samples 19,20.

Despite these technological advances in microfluidic immunomagnetic CTC capture systems, current microfluidic immunomagnetic CTC capture systems still face several limitations. Many platforms rely on complex device operation and require careful control of fluid conditions, which may affect reproducibility 8,14. In addition, most systems lack real-time visualization capability, making it difficult to directly monitor capture events and evaluate system performance during operation 17. Furthermore, flow instability caused by bubble formation remains a common issue that can disrupt microfluidic processes and reduce capture consistency. To address these limitations, the present study focuses on engineering integration to support more stable operation, enable real-time observation, and simplify workflow within a microfluidic immunomagnetic platform. Unlike conventional microfluidic immunomagnetic systems, the proposed platform integrates real-time visualization and flow stabilization, addressing key limitations related to system observability and operational robustness.

Rather than introducing a fundamentally new capture mechanism, this work focuses on engineering integration and operational simplification of immunomagnetic CTC capture within a microfluidic platform. The system is therefore positioned as a real-time observable and potentially live-cell-compatible enrichment platform. Further validation, including multi-marker capture strategies, quantitative purity analysis, and large-scale clinical studies, will be required to support future translational applications.

## 2. Materials and Methods

### 2.1 Microfluidic chip design, fabrication, and operation

**Figure 1:**
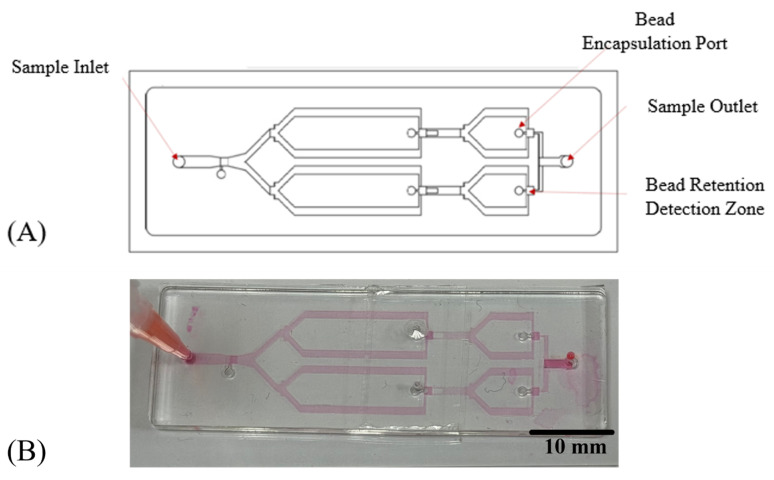
Microfluidic chip design and a fabricated device for magnetic bead-based cell capture. (A) Schematic illustration of the microfluidic chip layout. The device contains a single sample inlet, dual-branch microchannels for controlled fluid distribution, and bead-encapsulation ports positioned upstream of the bifurcation regions. Downstream, the microchannel converges toward the bead retention detection zone, where magnetic bead-cell complexes are trapped and analyzed. The processed fluid exits through the sample outlet after passing the detection region. (B) Photograph of the fabricated PDMS microfluidic chip bonded onto a glass substrate. The Y-shaped channel network and bead-trapping region are clearly visible, demonstrating successful molding and alignment of the microchannel architecture.

The microfluidic chip was designed as a multilayer platform composed of a bottom glass substrate and a polydimethylsiloxane (PDMS) microfluidic layer, integrating a top glass cover, a liquid chamber layer, a thick-film layer, and a thin-film layer (Figure 1). The liquid phase flowed between the glass and thin-film layers, whereas the gas phase was routed through the thick-film and liquid chamber layers without direct contact. The device incorporated a sample inlet, reagent storage reservoirs, and a waste reservoir. The sample inlet was used to introduce the blood sample pre-mixed with magnetic beads into the microfluidic channel. The reagent storage reservoirs served as temporary storage for buffer or washing solutions when required, enabling flexible fluid handling during operation. The waste reservoir collected the processed fluids after passing through the capture region, thereby preventing backflow and maintaining stable operation of the system. Pneumatic actuation was used to control the direction and flow rate of fluids within the microchannel network. The PDMS microfluidic layer contained a straight channel (1 mm wide × 18 mm long × 82 μm high) bonded to a glass substrate (25.4 mm × 38 mm × 1 mm).

### 2.2 Optimization of magnetic bead surface antibody conjugation

A biotin-streptavidin system was employed to functionalize the surface of streptavidin-coated magnetic beads. To determine the optimal biotin loading for bead functionalization, a series of biotin concentrations was evaluated. Saturation of the bead surface was achieved at 6 nmol biotin, and this condition was used for subsequent experiments.

Based on the optimized biotin loading, the biotinylated anti-CD326 (EpCAM) monoclonal antibody (clone 1B7; Invitrogen/eBioscience) was conjugated to streptavidin-coated magnetic beads under saturation conditions to generate EpCAM-functionalized beads for circulating tumor cell (CTC) capture.

### 2.3 Magnetic bead-antibody conjugation

Streptavidin-coated magnetic beads (Dynabeads™ M-280 Streptavidin, Invitrogen, Cat. No. 11205D) were used for bead functionalization. Before conjugation, the beads were gently resuspended and washed three times with phosphate-buffered saline (PBS, pH 7.4) to remove storage preservatives. Biotinylated anti-CD326 (EpCAM) monoclonal antibody (clone 1B7, eBioscience™, Cat. No. 13-9326-82) was diluted at 1:1000 and mixed with the magnetic bead suspension at a 1:1 (v/v) ratio of antibody solution to bead suspension, followed by incubation at room temperature for 60 min under gentle agitation to allow streptavidin-biotin binding. For capture experiments, approximately 10 µL of bead suspension was used per sample, corresponding to a bead-to-cell ratio of approximately 20:1 (beads:cells). After incubation, the antibody-conjugated beads were washed three times with PBS to remove unbound antibodies and subsequently resuspended in PBS or blocking buffer for further use. The functionalized beads were stored at 4 °C until use. Capture efficiency (%) was calculated as (number of captured cells / total number of spiked cells) × 100.

### 2.4 Sample processing and CTC capture procedure

CTC capture experiments were performed with whole blood spiked with cultured cancer cells, and blood samples were mixed with functionalized magnetic beads and injected into the microfluidic chip. Different flow rates (0.2-1.2 μL/s) were tested to optimize capture efficiency.

The CTC capture process was monitored in real time under a microscope, recording cell-bead interactions and capturing outcomes.

### 2.5 Cell culture

MCF-7 human breast cancer cells were purchased from ATCC (USA) and cultured in DMEM/F12 medium (HyClone, USA) supplemented with 10% fetal bovine serum (FBS; Sigma-Aldrich, USA) and 1% penicillin/streptomycin (Thermo Fisher Scientific, USA). Cells were maintained at 37 °C in a humidified incubator with 5% CO₂, and the culture medium was replaced every 2-3 days. Cells were passaged at approximately 70-80% confluence using standard trypsinization procedures.

### 2.6 Immunofluorescence staining and identification of CTCs

Captured cells were subjected to immunofluorescence staining for identification. After capture, the microfluidic chip was gently washed with phosphate-buffered saline (PBS) to remove unbound cells and debris. Cells retained within the microchannel were incubated with fluorescently labeled antibodies, including anti-CD326 (EpCAM)-FITC (clone G8.8, eBioscience™, Cat. No. 11-5791-82) and anti-CD45-PE (clone 30-F11, eBioscience™, Cat. No. 12-0451-82), for 30 min at room temperature in the dark. After incubation, the chip was washed three times with PBS to remove unbound antibodies. Nuclei were stained using Hoechst 33342 (Invitrogen, Cat. No. H1399) for 10 min at room temperature. Following staining, the chip was washed with PBS and immediately observed under a fluorescence microscope. Cells exhibiting Hoechst⁺/CD326⁺/CD45⁻ phenotypes were identified as putative circulating tumor cells (CTCs).

### 2.7 Statistical analysis

All quantitative data are presented as mean ± standard deviation (n = 3). Statistical analyses were performed using one-way analysis of variance (one-way ANOVA) to compare capture efficiencies under different experimental conditions. A p-value < 0.05 was considered statistically significant.

## 3. Results

### 3.1 Microfluidic chip architecture and pressure-regulated flow control

Figure 1 illustrates the overall design of the multilayer microfluidic chip and the integrated pneumatic control layout. During device operation, samples were introduced into the channel using a syringe-based microfluidic pumping system to generate controlled hydrodynamic interactions between flowing cells and bead structures within the microchannel. Under these flow conditions, cells rolled along the channel surface and collided with functionalized beads, enabling cell capture.

Fluid handling was precisely regulated using electronically controlled proportional valves coupled to a miniature pumping system. The applied pressure was maintained within a tunable range of 1-14 psi, allowing adjustment of flow conditions to optimize cell-bead interactions. Both positive and negative pressure modes were implemented to support infusion and withdrawal configurations, providing bidirectional flow control. The inlet and outlet were connected to a medium-filled platform, and continuous pumping ensured stable flow and reproducible operating conditions throughout the experiments.

### 3.2 Microchannel fluid dynamics and mixing effect

**Figure 2:**
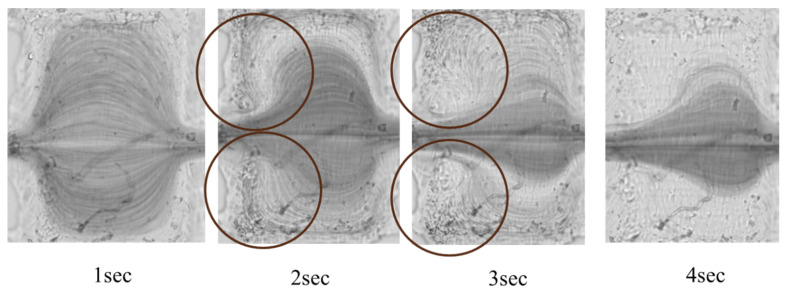
Flow-field visualization in the mixing region of the microfluidic chip. Tracer particles were used to visualize fluid behavior within the microchannel. Distinct ring-shaped vortices (highlighted by brown circles) were observed at specific geometric structures, where the flow bends and expands, generating localized vortex patterns.

To evaluate whether the microchannel design enhances fluid mixing and promotes cell-bead interactions, the mixing performance within the microchannels was assessed by tracer-particle visualization. As shown in Figure 2, special mixing structures embedded in the microchannel induced pronounced vortex flow as the fluid passed through these regions. Distinct ring-shaped vortex patterns were observed approximately 2-3 s after entering the structures, confirming effective vortex formation. This vortex-driven mixing is expected to enhance local fluid recirculation, thereby increasing the frequency of cell-magnetic bead encounters and improving cell-bead contact for subsequent capture. However, a quantitative evaluation of mixing efficiency was not performed in this study, and further analysis will be required to quantify the mixing performance.

### 3.3 Optimization of magnetic bead surface antibody conjugation

**Figure 3:**
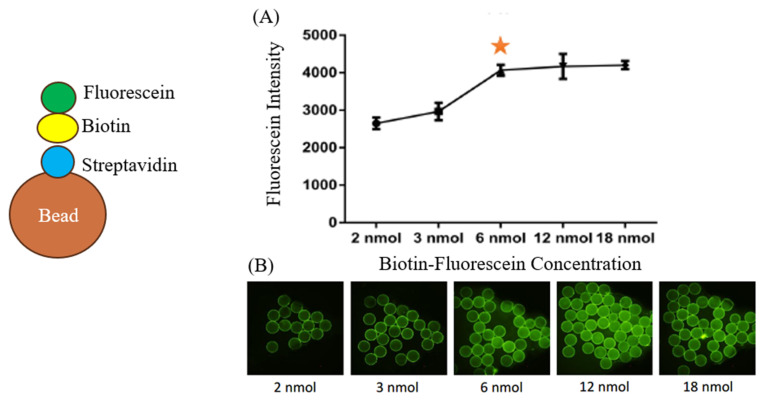
Optimization of biotin loading on streptavidin-coated magnetic beads. To determine the saturation level of available binding sites on streptavidin magnetic beads, increasing amounts of biotin (2-18 nmol) were incubated with the beads, followed by fluorescence quantification. (A) The fluorescence intensity increased progressively from 2 to 6 nmol, reaching a clear saturation plateau at 6 nmol, as indicated by the marked inflexion point. Further increases to 12 nmol and 18 nmol resulted in only minor changes, suggesting that the bead surface binding sites were largely saturated at 6 nmol. Data are presented as mean ± standard deviation (n = 3). Statistical analysis was performed using one-way ANOVA. (B) Representative fluorescence microscopy images confirm this trend. Beads treated with 2-3 nmol biotin exhibited weak and uneven fluorescence, whereas beads treated with 6 nmol showed strong and uniform fluorescence labelling. Little additional improvement was observed at 12 nmol and 18 nmol. Based on these results, 6 nmol was selected as the optimal biotin concentration for subsequent preparation of CD326-biotin (EpCAM)-functionalized magnetic beads.

As shown in Figure 3, biotin functionalization of the bead surface exhibited a concentration-dependent increase and reached saturation at 6 nmol, beyond which no further improvement in conjugation efficiency was observed. Therefore, 6 nmol biotin was selected as the optimal loading condition and applied for subsequent conjugation of biotinylated anti-EpCAM (CD326) antibodies to generate EpCAM-functionalized magnetic beads for downstream cell capture experiments (Figure 3).

### 3.4 Real-Time Observation of Cell Capture

**Figure 4:**
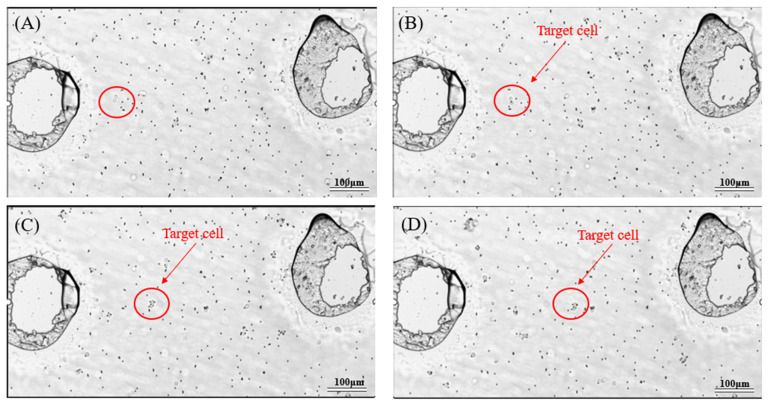
Microscopic observation of the dynamic interaction between target cells and magnetic beads during the capture process. The target cell is indicated by a red circle and arrow. Small dark particles surrounding the cell correspond to magnetic beads (approximately 2.8 μm in diameter). Scale bar: 100 μm.

Figure 4 illustrates the real-time capture behavior of target cells as they interact with antibody-functionalized magnetic beads under flow. As the cell moves from left to right within the microchannel, rolling and rotational motion are observed due to shear flow. Throughout this progression, the cell gradually establishes contact with nearby magnetic beads. Microscopic observation further confirmed the dynamic process of CTC-bead interactions. As shown in Figure 4, cells moved through the fluid, encountered beads, and gradually formed bead-cell complexes, from initial non-binding (Figure 4A) to multiple beads attached to a single cell (Figure 4D), confirming the system's capture mechanism.

### 3.5 Effect of flow rate on capture efficiency

**Figure 5:**
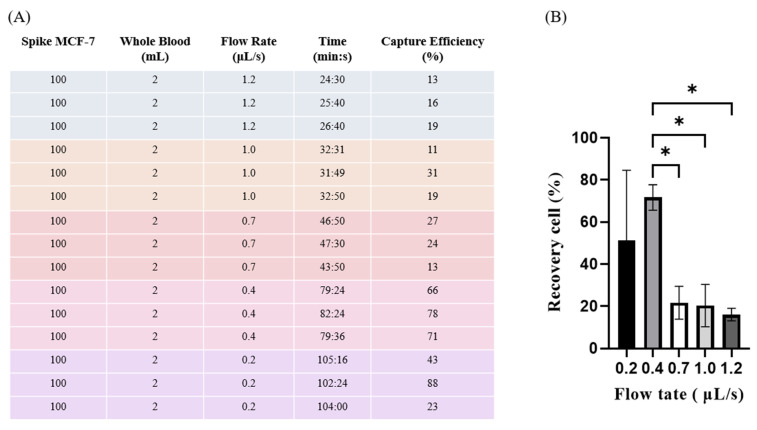
Capture efficiency of circulating tumor cells (CTCs) from whole blood at different flow rates. (A) Spike-in experiments were performed by adding 100 MCF-7 cells into 2 mL of whole blood and processing the samples at various flow rates (0.2-1.2 μL/s). Capture efficiency and processing time were recorded for each condition. The results show that a flow rate of 0.4 μL/s achieved the highest capture efficiency, with an average of 72% (range: 71-88%) and good consistency across repeated experiments, indicating consistent CTC capture performance in whole blood. (B) The bar graph summarizes the recovery rates at different flow rates. The 0.4 μL/s condition exhibited the highest average recovery (72%) among the tested flow rates. This optimal flow rate provides sufficient interaction time between cells and magnetic beads while maintaining an appropriate sample throughput, thereby improving capture efficiency. Error bars represent standard deviation (n = 3). Statistical significance was evaluated using one-way ANOVA (*p < 0.05).

To determine the optimal flow rate that balances capture efficiency and processing throughput, capture efficiency was evaluated at different flow rates (Figure 5). A relatively high capture efficiency was achieved at a flow rate of 0.4 μL/s. Lower flow rates reduced throughput, while higher flow rates were associated with reduced capture efficiency, likely due to decreased interaction between cells and beads, both negatively impacting capture. Although the 0.2 μL/s condition exhibited relatively high capture efficiency, the substantially longer processing time reduced overall throughput. Therefore, 0.4 μL/s was selected as the optimal condition by balancing capture efficiency and processing efficiency.

### 3.6 Evaluation of tumor cell capture performance in whole blood

**Figure 6:**
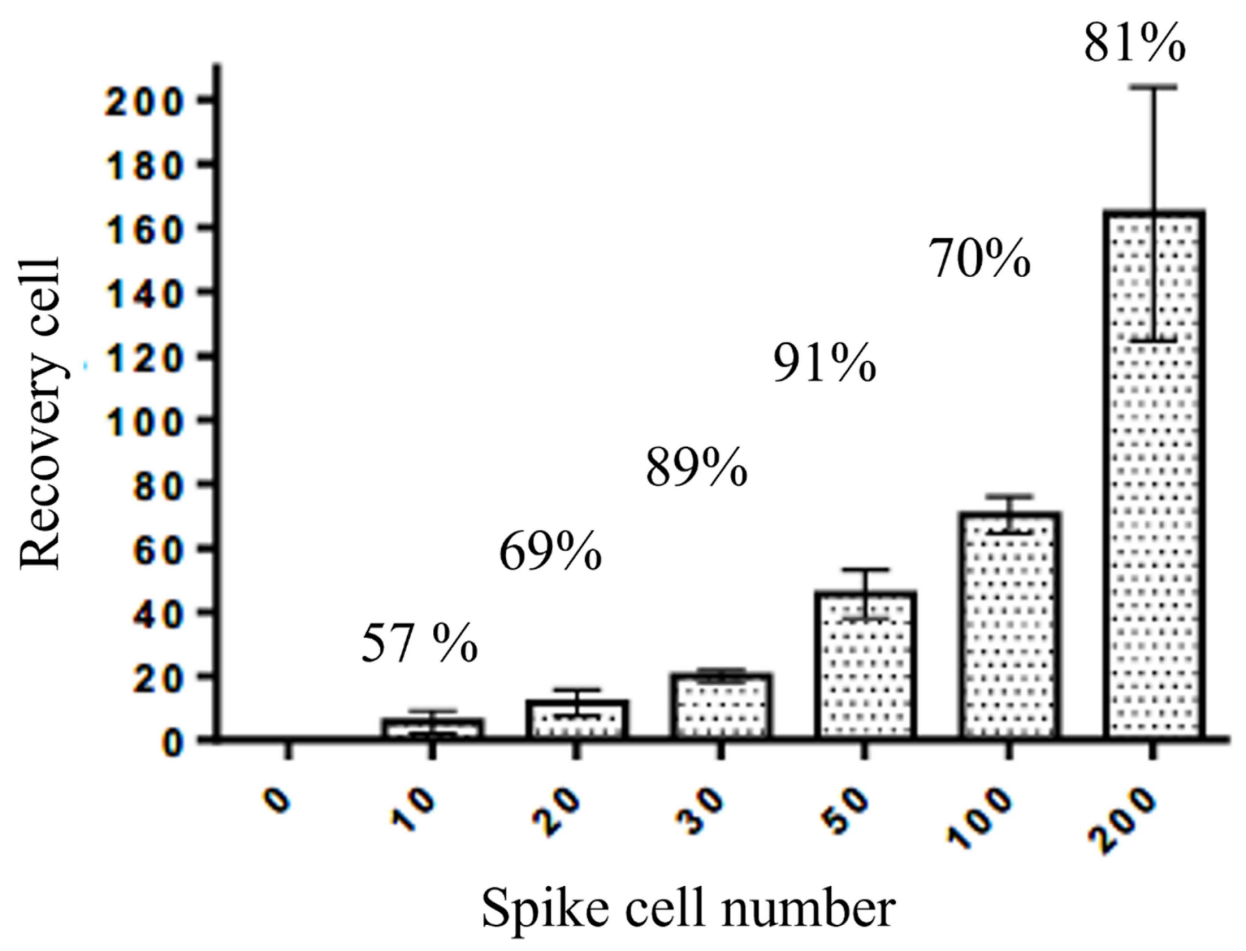
Capture performance of the platform in whole blood samples spiked with tumor cells. Different numbers of tumor cells (10-200 cells) were introduced into whole blood, and the recovered cells were quantified after processing. The number of recovered cells increased with increasing spike cell numbers, demonstrating a positive correlation between input and recovery. The capture efficiency ranged from approximately 57% to 91% across all tested conditions. Error bars represent standard deviation (n = 3).

To evaluate the capture performance under clinically relevant conditions, tumor cells were spiked into whole blood samples at varying cell numbers. As shown in Figure 6, the platform successfully recovered target cells across all tested conditions. The number of recovered cells increased with increasing spike cell numbers, demonstrating a positive correlation between input and recovery. The overall capture efficiency ranged from approximately 57% to 91%, indicating that the system maintains measurable capture performance in complex biological matrices such as whole blood. Notably, although relatively higher variability was observed at lower spike levels, the platform still demonstrated consistent recovery capability, suggesting its potential applicability for rare cell enrichment.

### 3.7 Identification of putative circulating tumor cells in patient blood samples

**Figure 7:**
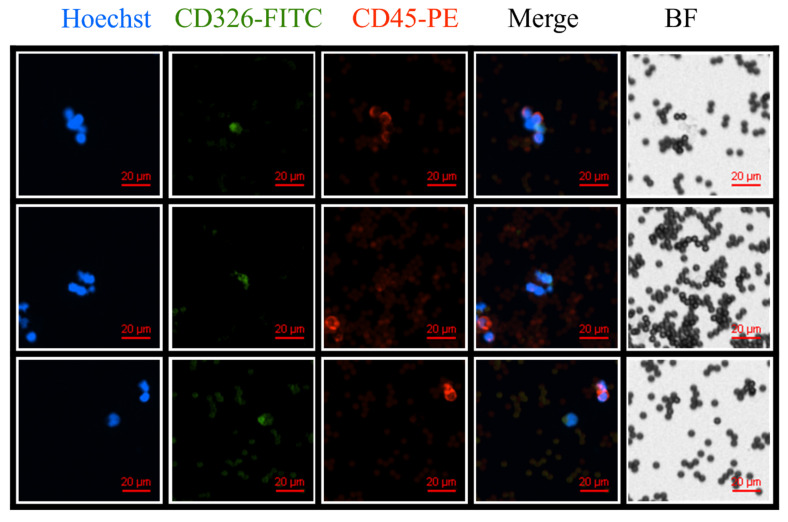
Identification of putative circulating tumor cells (CTCs) from patient blood samples. Blood samples from cancer patients were processed using the microfluidic platform, followed by immunofluorescence staining. Representative images show nuclei (Hoechst, blue), epithelial marker (CD326 (EpCAM)-FITC, green), and leukocyte marker (CD45-PE, red), along with merged and bright-field (BF) images. Cells exhibiting a Hoechst⁺/CD326⁺/CD45⁻ phenotype were identified, consistent with typical CTC characteristics. Scale bar: 20 μm.

To further evaluate the platform's applicability to clinically relevant samples, peripheral blood from cancer patients was processed using the microfluidic system. As shown in Figure 7, cells exhibiting characteristic CTC phenotypes were identified following capture, defined by positive nuclear staining (Hoechst), positive epithelial marker expression (CD326 (EpCAM)-FITC), and negative leukocyte marker expression (CD45-PE). These Hoechst⁺/CD326⁺/CD45⁻ cells were observed across multiple fields of view, showing morphological features consistent with tumor cells and clearly distinguishable from surrounding blood cells in both fluorescence and bright-field imaging. The results suggest the ability to isolate and enrich putative circulating tumor cells from patient-derived blood samples. Nevertheless, further molecular characterization may be required to confirm the tumor origin of the captured cells.

## 4. Discussion and Conclusions

In this study, we developed an integrated microfluidic immunomagnetic chip system for the enrichment of circulating tumor cells (CTCs) under continuous-flow conditions. A key feature of the proposed platform is its capability for rapid screening: as blood passes through the microchannels, antibody-functionalized magnetic beads enable rapid cell capture 15, allowing direct processing of whole blood without extensive preprocessing. The chip architecture further supports stable fluid handling while minimizing bubble-related interference 17,19, which contributes to improved operational robustness during prolonged runs. However, a direct quantitative comparison between the proposed gas-liquid separation structure and existing bubble-removal or flow-stabilization strategies was not performed in this study. Although a direct benchmarking study was not conducted, the observed performance falls within the range reported for comparable systems. Future work will include systematic benchmarking against established approaches.

The microchannel design appears to promote mixing and increase the frequency of cell-bead encounters 14, potentially increasing capture probability and enabling real-time microscopic observation of the capture process. This on-chip visualization provides direct confirmation of capture events and facilitates immediate assessment of system performance. With optimization of antibody conjugation parameters and a flow rate of 0.4 μL/s, the platform achieved capture efficiencies of approximately 57-91% in whole-blood spike-in experiments. Although a detailed theoretical analysis (e.g., shear stress or Peclet number) was not performed, the observed trends are consistent with reduced interaction time at higher flow rates and enhanced cell-bead interactions at lower flow rates.

These results are within the range of previously reported immunomagnetic microfluidic systems, which typically achieve recovery efficiencies in the range of 60-85% 13,14. However, it should be noted that the current study relies on MCF-7 cells, which represent an EpCAM-high epithelial model and may not fully reflect the heterogeneity of CTC populations in clinical samples 7,21.

A key limitation of this approach is the reliance on EpCAM-based capture, which may reduce the ability to isolate mesenchymal or EpCAM-low CTC subpopulations arising from epithelial-mesenchymal transition (EMT) 12,21. In addition, quantitative evaluation of capture purity, leukocyte contamination, and false-positive rates were not performed in this study and should be addressed in future work.

Although cells exhibiting Hoechst⁺/CD326⁺/CD45⁻ phenotypes were identified in patient-derived samples, this provides only preliminary evidence of clinical feasibility. Further validation using larger patient cohorts and molecular characterization will be required to confirm the tumor origin of the captured cells.

While the system is designed to minimize potential damage to cells during capture, no direct quantitative viability assays were conducted in this study. Therefore, the preservation of cell viability cannot be conclusively determined based on the current results. Future studies should include Live/Dead staining and proliferation assays to validate downstream applicability. In addition, the optimal flow rate (0.4 μL/s) represents a trade-off between capture efficiency and throughput. At this flow rate, processing a clinically relevant sample volume of 2 mL of blood would require approximately 1-1.5 hours. While this relatively low throughput may limit immediate clinical implementation, the selected condition prioritizes sufficient interaction time between cells and magnetic beads to improve capture efficiency. Future improvements may focus on increasing throughput through parallelization of microchannels or optimization of flow conditions without compromising capture performance.

Overall, this work positions the proposed system as an engineering-integrated, real-time-observable platform for CTC enrichment. Rather than introducing a fundamentally new capture mechanism, the study highlights the importance of system-level optimization in improving the stability and usability of microfluidic immunomagnetic technologies. With further validation and optimization, this system may provide a platform for liquid biopsy research and translational studies 20,21,22.
